# Comparison of pro- and anti-inflammatory responses in paired human primary airway epithelial cells and alveolar macrophages

**DOI:** 10.1186/s12931-018-0825-9

**Published:** 2018-06-25

**Authors:** Reem Al Mubarak, Nicole Roberts, Robert J. Mason, Scott Alper, Hong Wei Chu

**Affiliations:** 1 Department of Medicine, National Jewish Health, 1400 Jackson Street, Room A639, Denver, CO 80206 USA; 2 Department of Biomedical Research and Center for Genes, Environment, and Health, National Jewish Health, University of Colorado, 1400 Jackson Street, Denver, CO 80206 USA; 3 Department of Immunology and Microbiology, University of Colorado, 1400 Jackson Street, Denver, CO 80206 USA

**Keywords:** Tracheobronchial epithelial cells, Alveolar macrophages, Immune negative regulators, Inflammation, Toll-like receptor, Pathogen associated molecular patterns, Cigarette smoke

## Abstract

**Background:**

Airway epithelial cells and alveolar macrophages (AMs) are the first line of defense in the lung during infection. Toll-like receptor (TLR) agonists have been extensively used to define the regulation of inflammation in these cells. However, previous studies were performed in non-paired airway epithelial cells and AMs. The major goal of our study was to compare the pro- and anti-inflammatory responses of paired human primary airway epithelial cells and AMs to TLR3 and TLR4 agonists.

**Methods:**

Tracheobronchial epithelial cells (TBEC) and AMs from four smokers and four non-smokers without lung disease were cultured with or without Poly(I:C) (PIC) (a TLR3 agonist) or LPS (a TLR4 agonist) for 4, 24 and 48 h. The immune responses of paired cells were compared.

**Results:**

TBEC and AMs showed stronger pro-inflammatory cytokine (e.g., IL-8) responses to PIC and LPS, respectively. TLR3 and TLR4 mRNA levels were similar in non-stimulated TBEC and AMs. However, PIC stimulation in AMs led to sustained up-regulation of the immune negative regulators Tollip and A20, which may render AMs less sensitive to PIC stimulation than TBEC. Unlike AMs, TBEC did not increase NF-κB activation after LPS stimulation. Interestingly, smoking status was correlated with less TLR3 and IRAK-M expression in non-stimulated TBEC, but not in AMs. PIC-stimulated TBEC and LPS-stimulated AMs from smokers vs. non-smokers produced more IL-8. Finally, we show that expression of A20 and IRAK-M is strongly correlated in the two paired cell types.

**Conclusions:**

By using paired airway epithelial cells and AMs, this study reveals how these two critical types of lung cells respond to viral and bacterial pathogen associated molecular patterns, and provides rationale for modulating immune negative regulators to prevent excessive lung inflammation during respiratory infection.

**Electronic supplementary material:**

The online version of this article (10.1186/s12931-018-0825-9) contains supplementary material, which is available to authorized users.

## Background

Airway epithelial cells along with alveolar macrophages serve as the first line of host innate immune defense against airborne pathogens and other airborne environmental hazards [1]. These lung cells are able to recognize pathogen associated molecular patterns (PAMPs) using receptors that include the Toll-like receptor (TLR) family. TLR-mediated recognition of PAMPs leads to the activation of downstream signaling cascades, the subsequent activation of pro-inflammatory transcription factors including NF-κB, and ultimately the production of pro-inflammatory cytokines including CXCL8 (IL-8), CXCL10 (IP-10) and TNF-α. While this inflammatory response is important for combating the pathogens, inflammation must be appropriately regulated to prevent excessive inflammation and tissue damage. One regulatory mechanism that ensures that inflammation is self-limiting is the PAMP-mediated induction of negative regulators. Numerous negative regulators of TLR-mediated signaling have been identified including Toll-interacting protein (Tollip), TNF alpha-induced protein 3 or TNFAIP3 (A20) and interleukin-1 receptor-associated kinase 3 (IRAK-M). These negative regulators down-regulate the transcription and translation of TLR-induced genes during infection and inflammation [2]. Hosts are protected from hyper-inflammation and autoimmunity by the inhibitory effect of these negative regulators [2].

It has been proposed that airway epithelial cells and alveolar macrophages may respond similarly or differently to various microbes and microbial PAMPs [1, 3, 4]. However, few studies have been conducted that directly compare the response of these two cell types. Notably, negative regulators of TLR signaling pathways have not been previously investigated in airway epithelial cells and alveolar macrophages from the same human subject (paired cells) to clarify their effect on inflammatory responses.

In the current study, we used paired airway epithelial cells and alveolar macrophages from the same healthy donors to test the hypothesis that functional differences exist between airway epithelial cells and alveolar macrophages with respect to pro-inflammatory cytokine release after TLR stimulation. In particular, we tested if the response to TLR3 and TLR4 agonists differs in these two cell types and if this difference may be explained by altered expression of negative regulators of TLR signaling. We also explored the effect of smoking status in these paired cells on the immune response after TLR stimulation. A full understanding of how inflammation is regulated by these negative regulators in different host cell types will facilitate the design of new therapeutics to balance the beneficial and detrimental effects of inflammation in various lung diseases.

## Methods

### Materials

Bronchial epithelial cell growth medium (BEGM) with antibiotics was purchased from Lonza (Walkersville, MD). The BEGM was prepared following manufacturer’s guideline, which contained all the supplements (BPE, hEGF, epinephrine, transferrin, insulin, retinoic acid, triiodothyronine, GA) except hydrocortisone to avoid any inhibitory effect of corticosteroids on cell pro-inflammatory responses. RNA lysis buffer RLT was from Qiagen (Hilden, Germany). RIPA western lysis buffer was purchased from Thermo-Fisher Scientific (Waltham, MA). DMEM (high glucose) for making D10 (DMEM + 10% FBS + 1% Pen/Strep + 1% Amphotericin B + 1% L-Glutamine+ 0.5% Gentamicin) was from GE Life Sciences (Logan, UT). The nuclear extraction kit and TransAM NF-κB p65 assay kit were from Active Motif (Carlsbad, CA). IL-8, IP-10 and TNF-α ELISA kits were obtained from R&D systems (Minnieaplois, MN).

### Human donor information

To isolate human primary airway epithelial cells and alveolar macrophages, we obtained human lungs from de-identified organ donors whose lungs were not suitable for transplantation and were donated for medical research through the National Disease Research Interchange (Philadelphia, PA), the International Institute for the Advancement of Medicine (Edison, NJ), or Donor Alliance of Colorado. The Institutional Review Board (IRB) at National Jewish Health deemed this research as non-human subjects research. Donors were chosen based on lung function with a Pa_O2_/Fi_O2_ ratio of > 225, no history of clinical lung diseases, a chest radiograph indicating no infection, and a time on the ventilator of < 5 days. The sex, age, race, and smoking history of donors were variable and were not selection criteria.

### Isolation and culture of human tracheobronchial airway epithelial cells

**Fig. 1:**
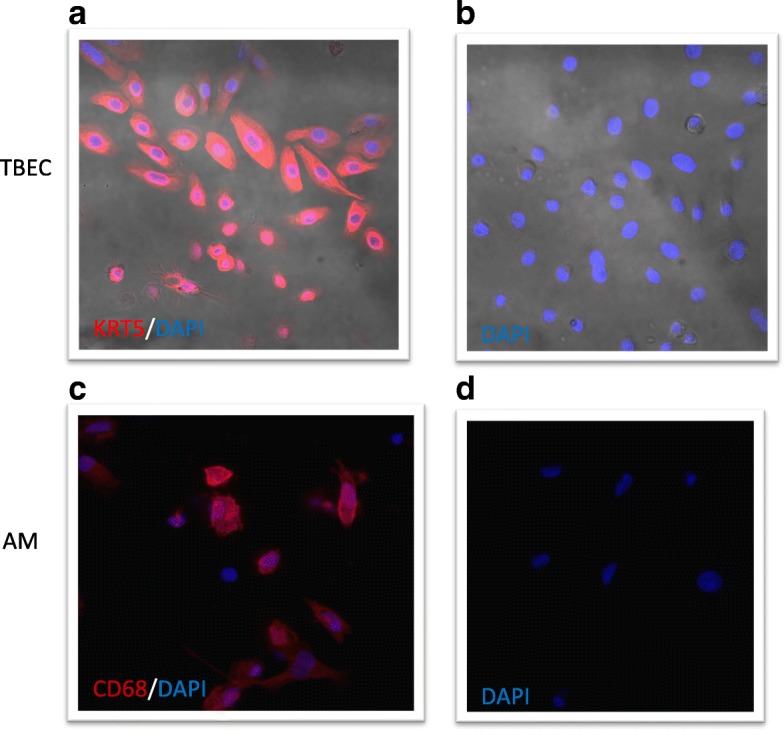
Characterization of primary human tracheobronchial epithelial cell (TBEC) and alveolar macrophages (AM) under submerged cultures. **a** TBEC showing features of basal cells with positive staining for cytokeratin-5 (KRT5 in red, DAPI in blue, 400X.); **b** Negative control for KRT5 stained with DAPI in blue (400X); **c** Primary alveolar macrophages (AM) stained positive for CD68 (red) and DAPI (blue, 400X); **d** Negative control for CD68, stained with DAPI in blue (400X)

To isolate tracheobronchial epithelial cells (TBEC), tracheal and main bronchial tissue was digested with 0.1% protease in DMEM overnight at 4 °C, and processed as previously described [5–7]. TBEC at passage 1 from the frozen stock were cultured and expanded in collagen-coated 60-mm tissue culture dishes in BEGM medium at 37 °C, 5% CO_2_. When the cells were 80% confluent, they were trypsinized and seeded onto 12-well plates for submerged culture. In our culture model (primary submerged culture), TBEC grown in a monolayer did not differentiate into the mucociliary phenotype, but showed the feature of basal cells expressing KRT5 (Fig. 1a, b).

### Submerged culture and stimulation of TBEC

TBEC at 1 × 10^5^ cells/well in BEGM media were seeded into 12-well plates. After 48 h, the medium was changed to refresh BEGM, and LPS (10 ng/ml) or Poly(I:C) (PIC) (1 μg/ml) was added. LPS was used to mimic bacterial infection as use of live bacteria could cause cell death, thus compromising data interpretation. We chose to use Poly I:C (PIC) as a dsRNA mimic of RNA viruses to broadly test the pro-inflammatory response in paired airway epithelial cells and alveolar macrophages. Given the varying susceptibility of the two types of cells to common respiratory viruses such as influenza A viruses and rhinoviruses, it would have been difficult to compare their responses to the live viruses. It has been reported that human AMs are less susceptible to infection by live influenza A viruses or rhinoviruses compared to epithelial cells [8]. PAMP doses were chosen following LPS and PIC dose response experiments; the lowest concentrations yielding a pro-inflammatory response in both cell types were chosen. The doses we chose were comparable to previous studies using LPS and PIC stimulation in cell culture experiments [9, 10]. The cells were harvested after 4, 24 and 48 h in RNA lysis buffer (RLT) or in RIPA buffer with protease and phosphatase inhibitors. The supernatants were collected and stored at − 80°C for ELISA.

### Isolation, culture, and stimulation of human alveolar macrophages

Bronchoalveolar lavage (BAL) was performed on the right middle lobe or lingula of the donor lungs by completely filling the lobe three times with balanced salt solution and EDTA, and then three times with the salt solution alone [11, 12]. After each instillation, lavage fluid was drained from the lung, collected, pooled, and centrifuged to obtain BAL cells including alveolar macrophages. The BAL cells were frozen in 90% FBS and 10% DMSO. Based on the protocols established by us [12, 13] and others [8, 14], macrophages were enriched from the BALF after lysis of the RBCs, and adhered onto the plastic surface in 12-well culture plates, and then washed to remove non-adherent cells. By using the CD68 immunofluorescent staining as shown in Fig. 1c, d and in our previous publication [13], nearly 99% of cells were positive for CD68.

Isolated alveolar macrophages were thawed and seeded into 12-well plates at densities of either 1 × 10^5^ cells/well or 5 × 10^5^ cells/well in D10 media at 37 °C in 5% CO_2_. After 48 h, the medium was changed to remove the non-adherent cells and treatments with LPS (10 ng/ml) or PIC (1 μg/ml) in D10 media were started on adhered AM. After 4, 24 and 48 h, the supernatants were collected and stored at − 80°C for ELISA. The cells were harvested at these same time points in RNA lysis buffer (RLT) or in RIPA buffer with protease and phosphatase inhibitors.

### Characterization of the TBEC cells and BAL macrophages with immunofluorescence

Primary paired TBEC and alveolar macrophages were cultured on coverslips. After 48 h, the TBEC were stained with an anti-cytokeratin antibody (KRT5) (Abcam, 1:500) and, AM were stained with an anti-CD68 antibody (Bioscience, 1:200) following a published protocol [6] Fig.1.

### NF-κB activity assay

Following the treatments of the paired cells as described above, nuclear proteins at each of the time points (4, 24, 48 h) were extracted using the Active Motif kit as per the manufacturer’s instructions. The extracted nuclear proteins (10 μg/condition) were tested for NF-κB p65 transcription factor activity using the TransAM NF-κB p65 kit following the manufacturer’s instructions. The data were expressed as optical density (OD) value.

### ELISA for human IL-8 (CXCL8), IP-10 (CXCL10) and TNF-α

IL-8, IP-10 and TNF-α protein levels were measured in cell supernatants using specific DuoSet ELISA kits (R&D Systems, Minneapolis, MN) as per the manufacturer’s instructions.

### RNA extraction and RT-PCR for human TLRs, Tollip, A20, IRAK-M, IL-8 and IP-10

RNA was extracted from cells stored in RLT using an RNeasy Plus kit (Qiagen). RNA was reverse transcribed using the High Capacity cDNA Reverse Transcription Kit (Applied Biosystems, California). Real-time PCR was performed on the CFX96 (Bio-Rad) using TaqMan gene expression assays from Applied Biosystems (Life Technologies, Foster City, CA, USA). An identical threshold cycle (Ct) was applied for each gene of interest (TLR3, TLR4, Tollip, A20, IRAK-M, IL-8 and IP-10). Relative mRNA expression and fold change levels were calculated using the delta Ct method for target genes and GAPDH. Target gene expression was normalized to GAPDH [15].

### Western blot analysis

Equal amounts of protein from samples with different treatments were separated by electrophoresis on 10% SDS-polyacrylamide gels. The proteins were then transferred onto nitrocellulose membranes and probed with either a goat polyclonal anti-Tollip antibody (sc27315), a mouse monoclonal anti-A20 antibody (sc69980) or a mouse anti-β-actin antibody (sc47778) (Santa Cruz Biotechnology, Inc.). Blots were then incubated with appropriate HRP-linked secondary antibodies and developed with the ECL Western blotting substrate. The blots were scanned using a Fotodyne imaging system, and densitometry was performed using the NIH Image-J software.

### Statistical analysis

Pairwise comparisons were performed using the Student’s *t*-test. For multiple comparisons, analysis of variance (ANOVA) or the Kruskal-Wallis test was used for normally distributed data or nonparametric data, respectively. False discovery rate (FDR) was used for correction (Benjamini and Hochberg). Single variable linear regression was used to test for an association between gene expression in AMs and TBECs. All statistical analyses and graphs were performed using GraphPad Prism software (GraphPad Software, La Jolla, CA, USA). *P*-values less than 0.05 were considered significant.

## Results

### Tracheobronchial epithelial cells (TBEC) and alveolar macrophages display different responses to poly(I:C) and LPS

The pro-inflammatory cytokines IL-8 (CXCL8) and IP-10 (CXCL10) were measured in the supernatants of cultured epithelial cells and macrophages from eight healthy subjects (four smokers and four non-smokers) as described in Table 1.

**Table 1:** Characteristics of Research Subjects

Subjects	Gender	Age (yrs)	Smoking status
1	Female	64	Smoker: 1pack/day × 43 years
2	Male	58	Smoker: < 1/2 pack/day × 20 years
3	Female	57	Smoker: < 1pack/day × 30 years
4	Male	58	Smoker: 1/2 pack/day × 37 years
5	Female	45	Non-smoker
6	Male	45	Non-smoker
7	Female	75	Non-smoker
8	Male	63	Non- smoker

**Fig. 2:**
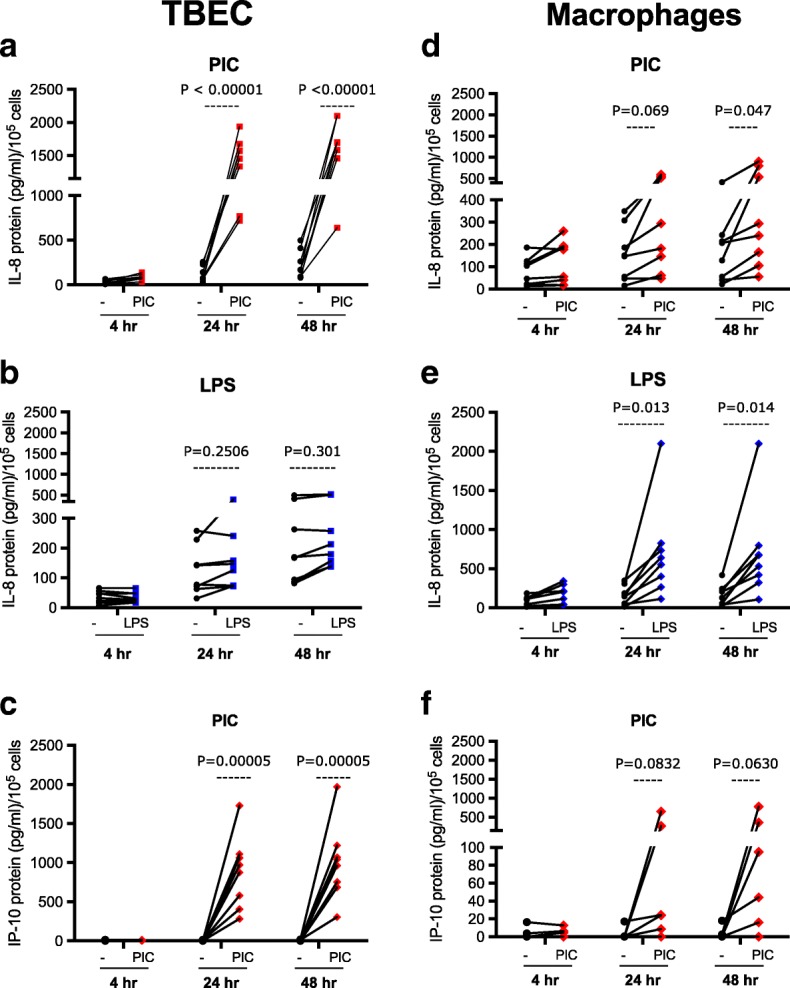
Human tracheobronchial epithelial cells (TBEC) and alveolar macrophages display different responses to different pathogen associated molecular patterns (PAMPs). IL-8 and IP-10 protein production in supernatants of cultured human TBEC (**a, b** and **c**) and cultured alveolar macrophages (**d, e** and **f**) in the absence (−) and presence of Poly(I:C) (PIC) or LPS treatment for 4, 24 and 48 h. *N* = 8 donor subjects

TBEC responded to PIC stimulation by producing IL-8 starting at 4 h, peaking at 24 h (*P* < 0.001), and then maintaining cytokine production at 48 h. (Fig. 2a). Similarly, TBEC increased IP-10 production after PIC stimulation (Fig. 2c). In contrast to PIC, LPS stimulation in TBEC did not significantly increase IL-8 production at any of the time points examined (Fig. 2b).

Alveolar macrophages responded to both PIC and LPS by producing IL-8, but the induction of IL-8 was stronger after LPS at 24 and 48 h (*P* = 0.01) (Fig. 2d and e). Alveolar macrophages also responded to PIC by producing IP-10 at 24 and 48 h, but the increase did not reach statistical significance (Fig. 2f).

**Fig. 3:**
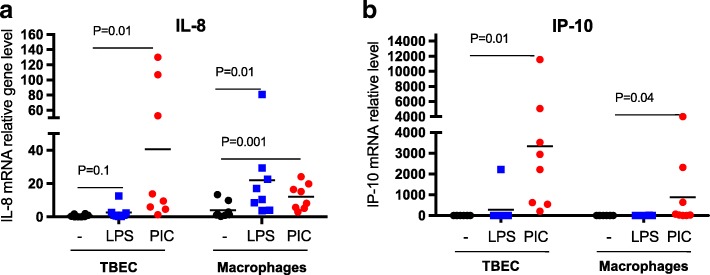
PAMP stimulation increases inflammatory cytokine mRNA levels in primary human lung cells. mRNA expression of IL-8 (**a**) and IP-10 (**b**) in human tracheobronchial epithelial cells (TBEC) and alveolar macrophages in the absence (−) and presence of LPS and Poly(I:C) (PIC) at 24 h. *N* = 8 donor subjects. The horizontal lines indicate the medians

Although the two cell types were initially seeded at the same cell density (1 × 10^5^/well), alveolar macrophages, unlike the TBEC, do not proliferate. As a complementary approach to monitoring cytokine protein levels produced by the two cell types, IL-8 and IP-10 mRNA levels were monitored at the peak time (24 h) of cytokine induction. Consistent with the protein data, in TBEC, IL-8 mRNA increased (> 20-fold) after PIC stimulation (*P* = 0.01), but did not increase after LPS stimulation (Fig. 3a). Macrophages showed a significant increase in IL-8 mRNA expression (> 15-fold) after LPS stimulation at 24 h (*P* = 0.01). Macrophages also increased IL-8 mRNA after PIC, but the induction level was about 50% of that in TBEC (Fig. 3a). Similar to IL-8, IP-10 mRNA levels after PIC stimulation at 24 h were higher in stimulated TBEC (about 3000-fold) than macrophages that showed about 1000-fold induction (Fig. 3b). Interestingly, IP-10 protein was not detectable after LPS stimulation in both alveolar macrophages and airway epithelial cells, and LPS did not significantelly induce IP-10 mRNA in both cell types (Fig. 3b). Together, our data suggest that TBEC are a better responder to PIC, and alveolar macrophages have a stronger response to LPS.

### Nuclear factor-kappaB (NF-κB) activation differs in paired TBEC and alveolar macrophages stimulated with Poly(I:C) or LPS

**Fig. 4:**
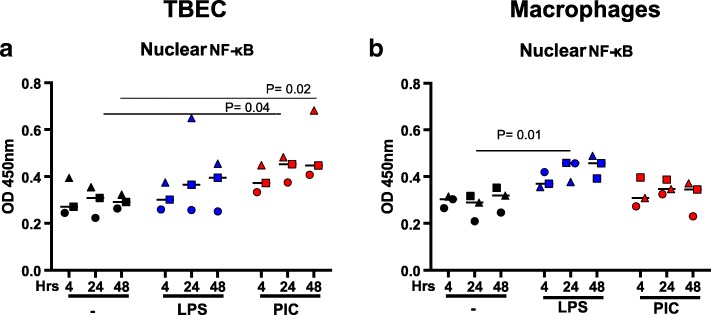
Differential activation of NF-κB in human tracheobronchial epithelial cells (TBEC) and alveolar macrophages in responses to PAMPs. NF-κB p65 levels were measured in nuclear extracts from paired TBEC (**a**) and alveolar macrophages (**b**) in the absence (−) and presence of treatments with LPS and Poly(I:C) (PIC) at 4, 24 and 48 h. *N* = 3 donor subjects

To determine the possible mechanism of varying pro-inflammatory cytokine responses of the paired airway epithelial cells and alveolar macrophages, NF-κB activity was measured with or without LPS or PIC stimulation. We chose to monitor NF-κB activation because NF-κB is activated by LPS and PIC and plays a key role in induction of pro-inflammatory cytokines. We monitored NF-κB activation by measuring NF-κB levels in nuclear extracts using an ELISA. Consistent with the IL-8 data, levels of NF-κB in the nuclei were increased in TBEC after stimulation with PIC (*P* < 0.05) at 24 and 48 h, but not LPS (Fig. 4a). On the other hand, LPS, but not PIC, significantly increased NF-κB activation in alveolar macrophages at 24 h (*P* = 0.01) (Fig. 4b).

### Levels of TLR4 and TLR3 expression do not explain the differences in IL-8 production and NF-κB activation in TBEC and alveolar macrophages

TLR4 and TLR3 are the respective receptors for LPS and PIC. Binding of TLRs to their ligands activates TLR signaling pathways, leading to activation of NF-κB and other transcriptional factors, and thus pro-inflammatory cytokine production [16]. In order to determine why TBEC and alveolar macrophages respond more strongly to PIC and LPS, respectively, we compared the baseline (untreated) expression of TLR3 and TLR4 in the two cell types. No significant differences of TLR3 or TLR4 mRNA expression were found between the two cell types (Additional file 1: Figure S1). This suggests that the differing responsiveness of TBEC and alveolar macrophages to PIC and LPS may be regulated by something other than TLR expression. One possibility that we explored below is that altered expression of negative regulators of TLR signaling may account for this difference.

### Differences in PAMP-mediated induction of negative regulators in TBEC

**Fig. 5:**
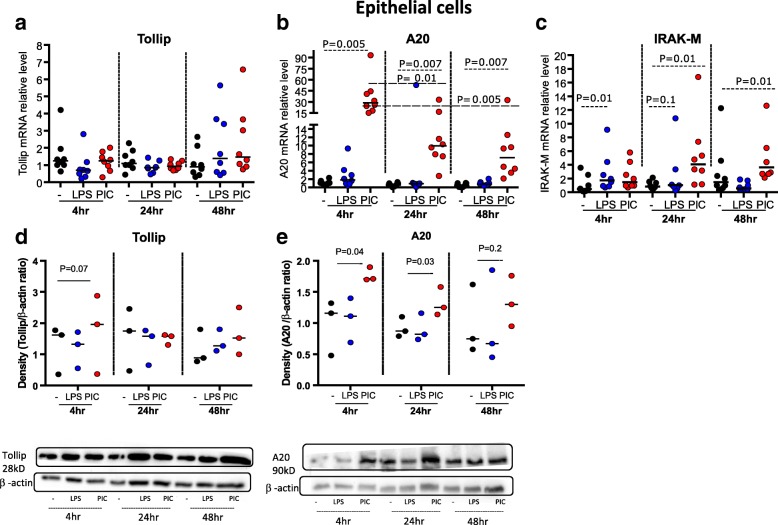
PAMP stimulation induces expression of negative regulators of TLR signaling in human tracheobronchial epithelial cells (TBEC). mRNA expression of negative regulators Tollip (**a**), A20 (**b**) and IRAK-M (**c**) in TBEC in the absence (−) and presence of LPS or Poly(I:C) (PIC) at 4, 24 and 48 h. *N* = 8 donor subjects. Median values are shown as horizontal lines. Densitometric analysis of Tollip (**d**) and A20 (**e**) western blots on lysates of TBEC in the absence (−) or presence of treatments with LPS, PIC at 4, 24 and 48 h. β-actin was included as a protein loading control to normalize Tollip or A20 expression. *N* = 3 donor subjects (additional blots in Additional file 1: Figure S2)

To potentially explain the different pro-inflammatory responses to PIC and LPS in TBEC and macrophages, we examined the expression of Tollip, A20 and IRAK-M, which are known to down-regulate TLR3 and TLR4 signaling pathways. In TBEC, there was no significant change in Tollip mRNA expression after LPS or PIC stimulation at all the time points measured (Fig. 5a). In contrast, the other two negative regulators did exhibit altered expression following PAMP challenge.

A20 mRNA levels were not significantly changed after LPS stimulation in TBEC. In contrast, PIC up-regulated A20 mRNA expression (*p* < 0.005) after 4, 24 and 48 h (Fig. 5b). A20 mRNA expression in TBEC was highest at 4 h after PIC stimulation and remained significantly increased, albeit at a lower level, 24 and 48 h after PIC challenge (Fig. 5b).

LPS treatment of TBEC did lead to a slight but not statistically significant increase in IRAK-M mRNA expression at 4 and 24 h after challenge but not at 48 h (Fig. 5c). In contrast, PIC treatment did not alter IRAK-M mRNA levels at the earliest time point but did significantly increase IRAK-M mRNA at the later 24 and 48 h time points (Fig. 5c). Thus, A20 expression was induced early after PIC challenge while IRAK-M expression was induced later after PIC challenge. Tollip expression was not altered at any time point examined.

We also examined Tollip and A20 protein levels by western blot in the subset of subjects (*n* = 3) with relatively abundant cells that allowed us to perform additional cell culture studies due to limited availability of alveolar macrophages in other subjects. In support of the mRNA data, Tollip protein levels were not changed significantly after LPS or PIC stimulation (Fig. 5d). Consistent with the mRNA data, A20 protein was induced significantly by PIC stimulation in TBEC with a significant increase at 4 and 24 h but not 48 h after challenge (Fig. 5e). Western blots for two other subjects are shown in Additional file 1: Figure S2 A-C.

### PAMP-mediated induction of negative regulators in alveolar macrophages overlaps but is distinct from that in TBEC

**Fig. 6:**
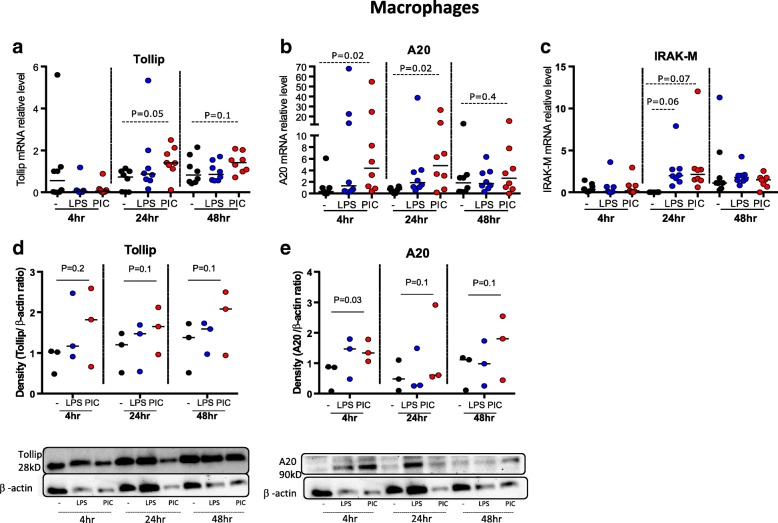
PAMP stimulation induces expression of negative regulators of TLR signaling in alveolar macrophages. mRNA expression of the negative regulators Tollip (**a**), A20 (**b**) and IRAK-M (**c**) in macrophages in the absence (−) and presence of LPS, Poly(I:C) (PIC) at 4, 24 and 48 h. *N* = 8 donor subjects. Median values are shown as horizontal lines. Densitometric analysis of Tollip (**d**) and A20 (**e**) western blots on lysates of macrophages in the absence (−) or presence of treatments with LPS, PIC at 4, 24 and 48 h. β-actin was included as a protein loading control to normalize Tollip or A20 expression. *N* = 3 donor subjects (additional blots in Additional file 1: Figure S2)

In the paired alveolar macrophages, Tollip mRNA was increased after 24 and 48 h of PIC treatment; in contrast, Tollip mRNA levels were not altered significantly by LPS treatment (Fig. 6a). Thus, Tollip expression is induced by PIC in alveolar macrophages but not in TBEC. A20 regulation in alveolar macrophages was similar to that in TBEC: PIC but not LPS induced A20 mRNA expression in macrophages at all the time points (4, 24 and 48 h) (Fig. 6b). IRAK-M mRNA marginally increased at 24 h post LPS and PIC stimulation but returned to baseline levels by 48 h (Fig. 6c).

To confirm the mRNA expression data, we also monitored Tollip and A20 protein levels in the macrophages after LPS and PIC stimulation. We found that both Tollip and A20 protein levels were increased after PIC challenge, but this only reached statistical significance for A20 after 4 h of challenge (Fig. 6d, e). Western blots for two other subjects are shown in Additional file 1: Figure S2 A-C. The more robust effects at the mRNA level rather than at the protein level may be due to the larger variability observed in the protein data.

Together, the above data showed that after PIC stimulation, Tollip, A20 and IRAK-M were all up-regulated in alveolar macrophage, while only A20 and IRAK-M were increased in TBEC. Moreover, there were temporal differences in this induction. LPS, on the other hand, did not increase production of any of these three negative regulators in either cell type.

### Correlation in expression of negative regulators between paired airway epithelial cells and alveolar macrophages

**Fig. 7:**
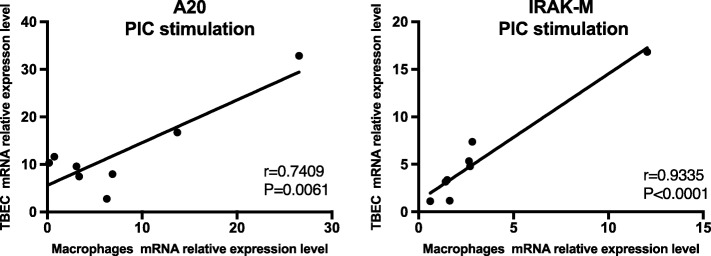
Expression of the negative regulators A20 and IRAK-M are strongly correlated between paired human tracheobronchial epithelial cells (TBEC) and alveolar macrophage samples from the same individual donors. A20 and IRAK-M mRNA expression showed significant correlation between the two cell types after 24 h of PIC stimulation. *N* = 8 donor subjects

We and others have observed substantial variability in the response of AMs and TBECs from different donors to PAMP stimulation [3, 4]. However, to our knowledge, prior studies have not addressed if AMs and TBECs from individual donors acted similarly regarding their responses to PAMPs. We therefore analyzed our cytokine, TLR, and negative regulator expression data to see if expression correlated in the two cell types from individual donors. We found that IL-8, IP-10, TLR3 and TLR4 expression in these paired analyses did not correlate (Additional file 1: Table S1 with *p* values). In contrast, the negative regulators A20 and IRAK-M showed striking positive correlations after 24 h of PIC stimulation (*P* < 0.01) (Fig. 7).

### The impact of smoking status on TLR expression and pro-inflammatory responses to PIC and LPS

To determine if smoking status alters the regulation of inflammation in TBEC or alveolar macrophages, we compared cytokine production following PIC or LPS stimulation, the levels of TLR3 and TLR4, and the levels of negative regulators in cells from donors with or without smoking history.

**Fig. 8:**
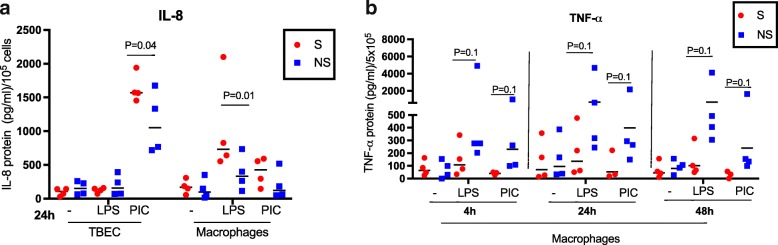
Smoking history alters IL-8 expressionat the protein level in human tracheobronchial epithelial cells (TBEC) and macrophages, and decreases TNF-α production in macrophages treated with LPS. **a** IL-8 production was measured in TBEC and alveolar macrophages in the absence (−) or presence of LPS or poly(I:C) (PIC) at 24 h, and compared between smokers (S, *n* = 4) and non-smokers (NS, *n* = 4). These data are a re-analysis of the data displayed in Fig. 3a. There is significant induction of IL-8 in smokers’ TBEC after PIC stimulation, and in smokers’ macrophages after LPS stimulation. **b** TNF-α production in supernatants of cultured alveolar macrophages. The cells from smokers (S, *n* = 4) and non-smokers (NS, *n* = 4) were treated in the absence (−) or presence of LPS or PIC for 4, 24 and 48 h. NS trend to have higher levels of TNF-α at all the time points (*P* = 0.1)

PIC treatment induced more IL-8 in TBEC from smokers than non-smokers at the protein (Fig. 8a) and mRNA levels (Additional file 1: Figure S3). Likewise, LPS treatment induced more IL-8 in alveolar macrophages from smokers than non-smokers at the protein (Fig. 8a) and mRNA levels. (Additional file 1: Figure S3) Thus, smoking enhanced IL-8 production in both cell types. While smoking enhanced IL-8 production in both cell types, this effect was not observed for other pro-inflammatory cytokines. Smoking weakened LPS and PIC-induced TNF-α production in alveolar macrophages (Fig. 8b), and smoking did not significantly alter IP-10 production in either PIC-stimulated TBEC or alveolar macrophages (Additional file 1: Figure S3). Although TNF-α was increased in supernatants of alveolar macrophages stimulated with TLR agonists, it was not detectable in airway epithelial cell supernatants under any conditions. Thus, we cannot compare the production of TNF-α between alveolar macrophages and airway epithelial cells stimulated with TLR agonists.

**Fig. 9:**
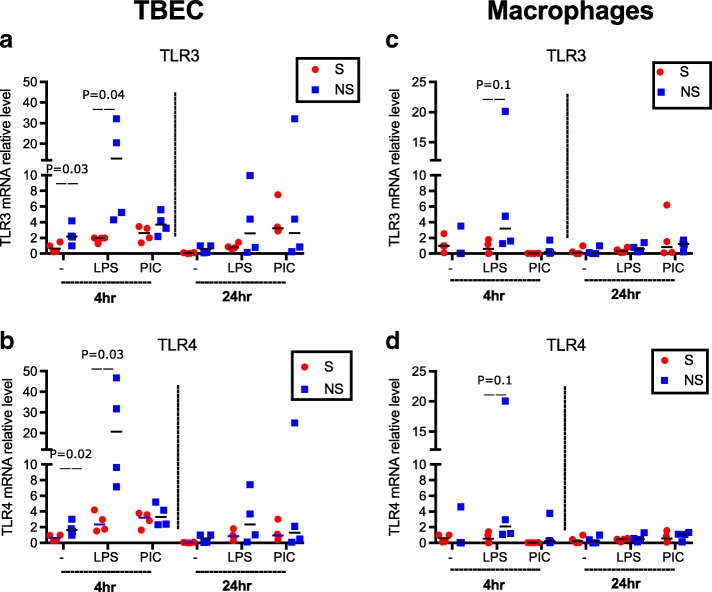
Smoking history alters TLR expression in human tracheobronchial epithelial cells (TBEC) but not macrophages. TLR3 and TLR4 mRNA expression in TBEC (**a** and **b**) and alveolar macrophages (**c** and **d**) that were not treated, or treated with LPS or Poly(I:C) (PIC) for 4 and 24 h. *N* = 8 donor subjects including four smokers (S) and four non-smokers (NS)

To determine how smoking altered the inflammatory response, we monitored expression of TLR3 and TLR4 and negative TLR regulators. Unstimulated TBEC from non-smokers had greater mRNA expression of TLR3 and TLR4 than TBEC from smokers (*P* < 0.05) (Fig. 9a). Moreover, after LPS stimulation, TLR3 and TLR4 mRNA levels significantly increased at 4 h in TBEC from non-smokers compared with smokers (*P* < 0.05) (Fig. 9a, b). In contrast, PIC did not alter TLR3 or TLR4 mRNA levels significantly in either smokers or non-smokers (Fig. 9a, b). In alveolar macrophages, TLR3 and TLR4 mRNA levels were not altered significantly by smoking status in the absence or presence of PAMP stimulation (Fig. 9c, d), although there was a weak trend towards more TLR3 and TLR4 at 4 h after LPS stimulation (Fig. 9c, d). Thus, changes in TLR expression could not account for all the effect of smoking on inflammatory cytokine production in these two cell types.

We also measured the levels of the negative regulators in non-stimulated TBEC and macrophages from smokers and non-smokers. No differences in Tollip and A20 expression were found between cells from the smokers and non-smokers (data not shown). However, IRAK-M mRNA expression in TBEC was lower in the smokers than the non-smokers at 24 h (*P* = 0.01) (Additional file 1: Figure S4).

## Discussion

The present study leverages the use of paired airway epithelial cells and alveolar macrophages from the same donors in order to clearly demonstrate how these two types of critical innate immune cells respond to two major TLR agonists (PIC and LPS) that are relevant to bacterial and viral lung infections.

Paired TBEC and macrophages showed differential immune responses to PIC and LPS stimulation. While TBEC have a greater pro-inflammatory response (IL-8, IP-10) to the TLR3 agonist PIC, alveolar macrophages are a stronger responder to LPS, a TLR4 agonist. These differential responses at the inflammatory cytokine level were likely driven by differences in NF-κB activation in the two cell types. In the TBEC, NF-κB activity was higher after PIC stimulation; in contrast, macrophages showed higher NF-κB activity after LPS stimulation.

In order to understand the mechanisms behind this differential immune response in the paired cells after LPS and PIC stimulation, we monitored their TLR3 and TLR4 expression. There were no significant differences in the mRNA expression of TLR3 or TLR4 between the paired cells that were not stimulated with either PIC or LPS. Thus baseline expression levels of TLR3 and TLR4 may not be responsible for the differential cell type-specific responses to TLR agonists. Because of the limited availability of primary human alveolar macrophages, we analyzed TLR mRNA but not protein expression. Previous studies indicate that mRNA expression of TLRs such as TLR3 in epithelial cells is consistent with protein expression determined using flow cytometry [17], suggesting that TLR mRNA is a reasonable surrogate for TLR protein levels. Nevertheless, this is one limitation of our study. It has been found that TLR4 in AEC is normally localized in the endosomal compartment, but is translocated to the cell surface to recognize pathogens or environmental LPS exposure [18, 19]. In contrast, macrophages express TLR4 primarily at the cell surface membrane [19]. While TLR3 in macrophages is usually observed intracellularly, it is found on the cell surface as well as in the cytoplasm of AEC [17, 20]. The different localization of TLR3 and TLR4 in various types of lung cells may provide an additional explanation for their different responses to TLR agonists.

Despite the fact that the two cell types showed no differences in TLR4 and TLR3 expression following LPS or PIC stimulation, their NF-κB activity and subsequent inflammatory cytokine production differed. These data suggest the involvement of other mechanisms or regulators in the differential activation of epithelial cells and macrophages exposed to TLR agonists. One possibility that we explored was expression of negative regulators of TLR signaling.

The literature suggests that TLR-induced expression of immune negative regulators represents a critical negative feedback mechanism to prevent excessive TLR signaling. These negative regulators function at multiple levels in the TLR signaling pathway ranging from inhibition of receptor complex protein formation to NF-κB activation [21–24]. Previous studies have elucidated the role of immune negative regulators such as A20, Tollip and IRAK-M in the regulation of LPS-mediated pro-inflammatory responses of unpaired airway epithelial cells and lung macrophages. For example, A20 was found to attenuate airway epithelial cell responses to TLR2 and TLR4 agonists [25, 26] as well as endotoxin tolerance in macrophages [27]. The role of A20 in antiviral responses has not been well studied. To the best of our knowledge, our study is the first one to investigate the time course of immune negative regulator expression in the response of paired lung cells (AM, AEC) to the viral mimic PIC as well as LPS. This allowed us to determine the potential mechanisms of differential responses of airway epithelial cells and alveolar macrophages to various TLR agonists.

We found that the expression of these negative regulators differed with respect to time and cell type. Alveolar macrophages up-regulated multiple immune negative regulators including Tollip, A20 and IRAK-M after PIC stimulation. While A20 mRNA and protein were induced rapidly after PIC stimulation, Tollip and IRAK-M were induced at later times. Importantly, A20 induction was maintained throughout the entire 48 h post PIC stimulation. In contrast, TBEC showed significant induction of A20 only at the earliest time point after PIC stimulation, but at later times (24 and 48 h), A20 expression significantly declined. Moreover, unlike macrophages, PIC-stimulated TBEC did not significantly change the expression of Tollip. We speculate that the lack of PIC-induced Tollip induction coupled with the transient A20 induction in TBEC may contribute to their more robust pro-inflammatory responses than the alveolar macrophages. The early induction and late reduction of A20 in PIC-stimulated TBEC are consistent with a previous publication performed by Gu et al. in the human cytomegalovirus (HCMV) infection model [28]. Together, our data indicate the possibility that these immune negative regulators could affect the differential response of various types of lung immune cells to PAMP stimulation. Future functional studies using the RNA interference may further our understanding of these immune negative regulators in modulating TLR agonist-mediated pro-inflammatory responses in paired alveolar macrophages and airway epithelial cells.

The design of our current study was also aimed to address the effect of smoking status on pro-inflammatory response in paired TBEC and alveolar macrophages as smoking has been linked to changes in the immune response in airway cells [29, 30]. Macrophages from smoking subjects produced less TNF-α but more IL-8 after LPS stimulation. However, in TBEC, IL-8 induction was significantly higher in smoker’s cells than non-smoker’s cells after PIC but not LPS stimulation. One possible explanation for these differences in the cells previously exposed to cigarette smoke was the difference in TLR3 and TLR4 expression. As observed previously [31–34], smoking down-regulated TLR3 and TLR4 expression in TBEC with and without stimulation. The down-regulation of TLRs by smoking was consistent with the decreased TNF-α and IP-10 production in the smokers, but not the increased IL-8 production following stimulation. Many studies have shown that cigarette smoking can inhibit inflammatory cytokine production (TNF-α, IL-6) and host defense responses (IFN-β, IP-10) in response to TLR stimulation [30, 35, 36]. However, there are discrepancies in the literature regarding the effect of smoking on IL-8 production. While some studies have demonstrated a reduction in IL-8 production or no effect from smoking [37], other studies have reported increased production of IL-8 and other neutrophil chemokines from smoker’s airway cells after TLR agonist stimulation [33, 34, 38]. The signal transduction mechanisms controlling TNF-α and IL-8 production appear to be different in the alveolar macrophage. In macrophages, smoking has been shown to activate p38 MAPK that controls transcription, stabilization of mRNA and secretion of IL-8 but not TNF-α [34].

The induction of the negative regulators observed in our study may also contribute to the differences in cytokine production in cells from smokers compared to non-smokers. Our data showed that smoking decreased expression of IRAK-M without stimulation in TBEC, which is consistent with the increased IL-8 production that we observed in smokers.

A significant strength of our study was our bank of paired TBECs and AMs, which allowed us to determine if the response to TLR agonists in different cell types correlated within individual donors. We did not observe any correlation in inflammatory cytokine production between the two paired cell types. Thus, the robustness of an individual’s immune response in one key lung innate immune cell type did not correlate with the robustness of that response in the other cell type. Despite this observation, we did identify a very strong correlation in PIC-induced expression of negative regulators in the two cell types. Consistent with the mRNA data, in preliminary studies on a small number of samples, we also observed a trend of positive correlations of A20 and Tollip protein levels between the two cell types (data not shown). This indicates that an individual’s ability to restrain inflammation in the lung may extend to multiple cell types.

One limitation of our study is that cultured cells may not maintain the phenotype of the in vivo cells, including the loss of the full differentiation status. Unfortunately, this is an inherent issue for every cell culture study. Nevertheless, one advantage of the cell culture model is to allow us to quantitatively analyze the impact of multiple TLR agonists. In the future study, we plan to validate our results by using other culture methods such as air-liquid interface to maintain the differentiation status of airway epithelial cells. Also, it will be interesting in the future to study how airway epithelial cells and alveolar macrophages cooperate in the effective lung defense against any environmental hazards using the co-culture model.

## Conclusions

**Fig. 10:**
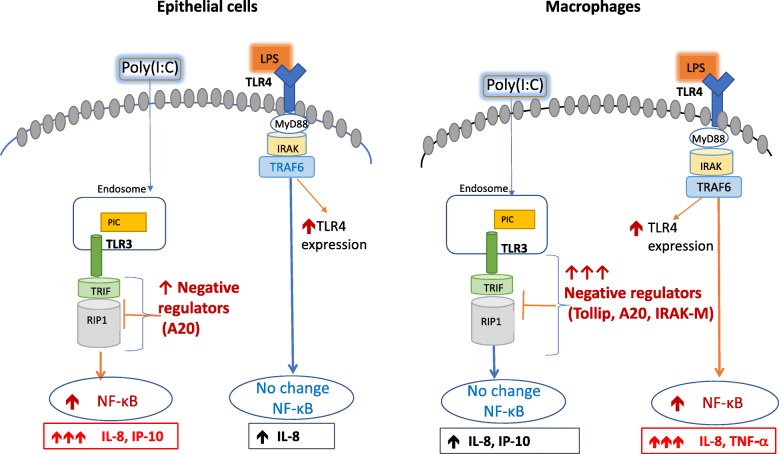
Proposed immune regulatory mechanisms of paired airway epithelial cells and lung macrophages in responses to Poly(I:C) and LPS stimulation

In summary, we find that TBEC and alveolar macrophages exhibit different responses to different TLR agonists. While these cells did not exhibit different levels of TLR mRNAs, they did exhibit different expression of negative regulators of TLR signaling, which could impact the extent of the immune response in these two cell types. Addtionally, our data suggest a mild impact of smoking status on the pro- (e.g., IL-8 and IP-10) and anti-inflammatory (e.g., A20 and Tollip) responses at the baseline and/or after TLR agonist stimulation. We propose the involvement of potential pathways in regulating the different responses of airway epithelial cells and alveolar macrophages to TLR agonists in Fig. 10. Understanding the role of immune negative regulators in primary human lung cells may provide new and promising therapeutic strategies to control pulmonary inflammation and infection.

## Additional file

Additional file 1: **Figure S1.** Comparison of baseline (untreated) expression of TLR3 and TLR4 in human tracheobronchial epithelial cells (TBEC) and alveolar macrophages (AM). No significant differences of TLR3 or TLR4 mRNA expression were found between the two cell types. **Figure S2.** Detection of A20, Tollip in human tracheobronchial epithelial cells (TBEC) and alveolar macrophages before and after PAMP stimulation at different time points. Western blot showing A20, Tollip, and β-actin proteins from TBEC and alveolar macrophages before (−) and after treatments with LPS, Poly(I:C) (PIC) for 4, 24 and 48 h (*N* = 3). These blots were also used for desitometry in Figs. 5 and 6. **Figure S3.** Comparison of IL-8 and IP-10 expression in smokers and non-smokers human tracheobronchial epithelial cells (TBEC) and macrophages. mRNA expression of IL-8 and IP-10 in TBEC and alveolar macrophages in the absence (−) or presence of LPS or Poly(I:C) (PIC) at 24 h was compared between smokers (S, *n* = 4) and non-smokers (NS, *n* = 4). These data are a re-analysis of the data displayed in Fig. 2. **Figure S4.** The effect of smoking status on IRAK-M expression. IRAK-M mRNA expression was examined after 24 h of culture in non-stimulated human tracheobronchial epithelial cells from smokers (S, *n* = 4) and non-smokers (NS, *n* = 4). NS have higher IRAK-M expression than S. **Table S1.** Correlation analysis between paired airway epithelial cells and alveolar macrophages from the same donors with *P*-values. (PPTX 367 kb)

## Abbreviations

AMs: Alveolar macrophages
BAL: Bronchoalveolar lavage
IRAK-M: Interleukin-1 receptor-associated kinase 3
LPS: Lipopolysaccharide
PAMPs: Pathogen associated molecular patterns
PIC: Poly(I:C)
TBEC: Tracheobronchial epithelial cells
TLR: Toll-like receptor
TNF-α: Tumor necrosis factor-alpha
TNFAIP3 (A20): TNF alpha-induced protein 3
Tollip: Toll-interacting protein
