# Supplementary material for: Comparison of pro- and anti-inflammatory responses in paired human primary airway epithelial cells and alveolar macrophages
Source: Respir Res. 2018 Jun 25;19:126. doi: 10.1186/s12931-018-0825-9 (PMC6020222; doi:10.1186/s12931-018-0825-9)
Supplement: Supplementary file 1 — Figure S1. Comparison of baseline (untreated) expression of TLR3 and TLR4 in human tracheobronchial epithelial cells (TBEC) and alveolar macrophages (AM). No significant differences of TLR3 or TLR4 mRNA expression were found between the two cell types. Figure S2. Detection of A20, Tollip in human tracheobronchial epithelial cells (TBEC) and alveolar macrophages before and after PAMP stimulation at different time points. Western blot showing A20, Tollip, and β-actin proteins from TBEC and alveolar macrophages before (−) and after treatments with LPS, Poly(I:C) (PIC) for 4, 24 and 48 h (N = 3). These blots were also used for desitometry in Figs. 5 and 6. Figure S3. Comparison of IL-8 and IP-10 expression in smokers and non-smokers human tracheobronchial epithelial cells (TBEC) and macrophages. mRNA expression of IL-8 and IP-10 in TBEC and alveolar macrophages in the absence (−) or presence of LPS or Poly(I:C) (PIC) at 24 h was compared between smokers (S, n = 4) and non-smokers (NS, n = 4). These data are a re-analysis of the data displayed in Fig. 2. Figure S4. The effect of smoking status on IRAK-M expression. IRAK-M mRNA expression was examined after 24 h of culture in non-stimulated human tracheobronchial epithelial cells from smokers (S, n = 4) and non-smokers (NS, n = 4). NS have higher IRAK-M expression than S. Table S1. Correlation analysis between paired airway epithelial cells and alveolar macrophages from the same donors with P-values. (PPTX 367 kb) [file 12931_2018_825_MOESM1_ESM.pptx]

## Slide 1
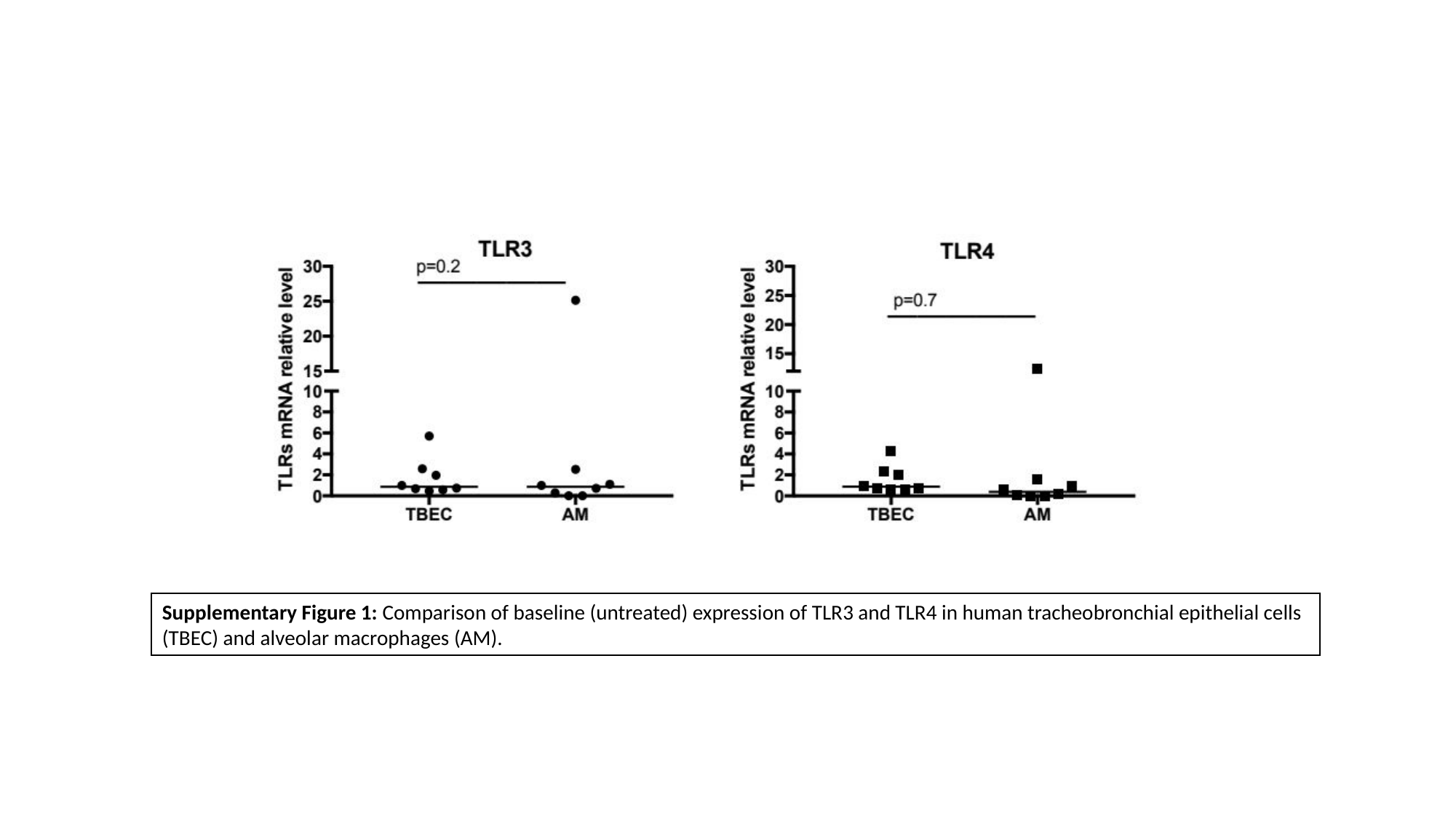

Supplementary Figure 1: Comparison of baseline (untreated) expression of TLR3 and TLR4 in human tracheobronchial epithelial cells (TBEC) and alveolar macrophages (AM).

## Slide 2
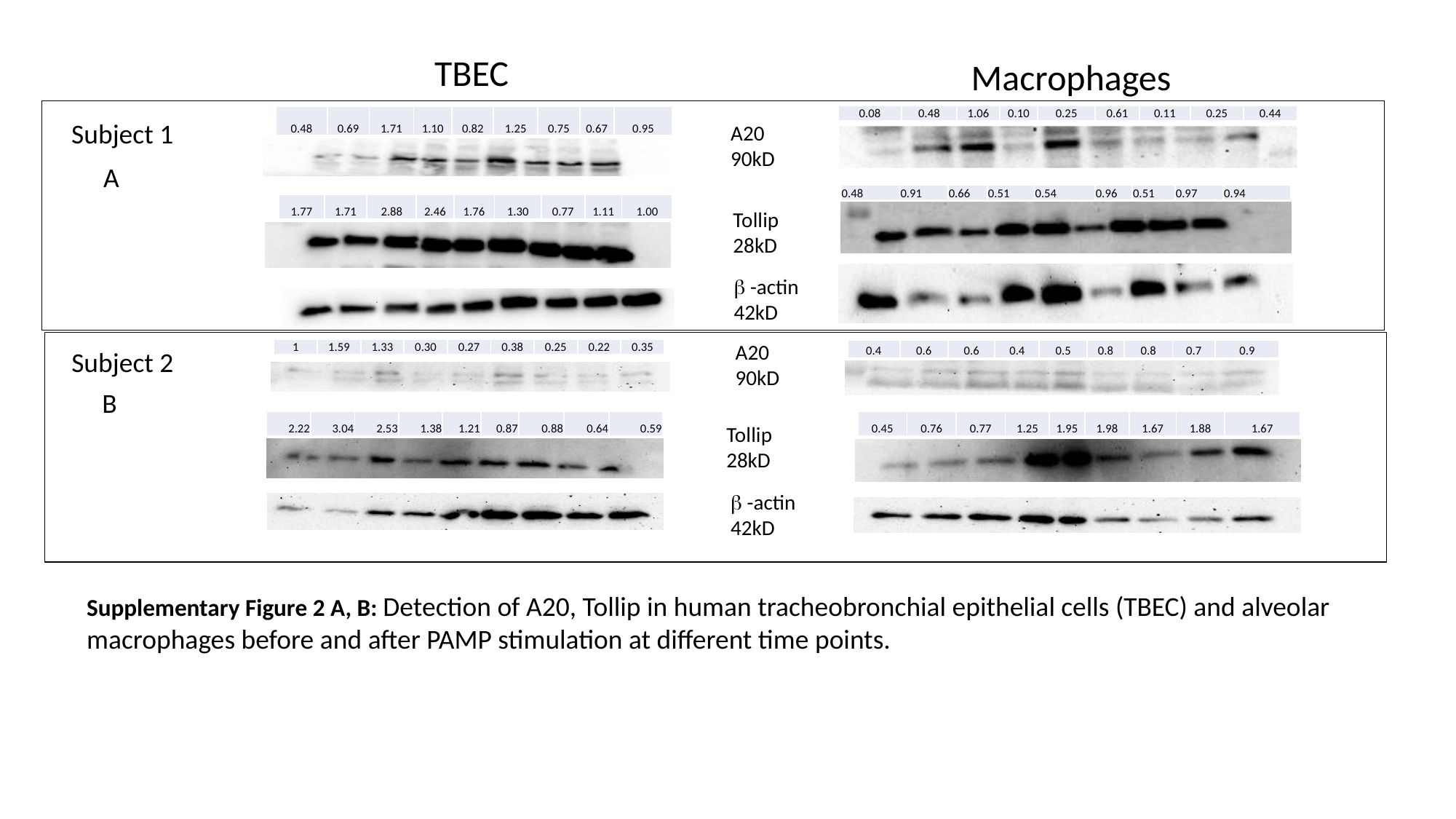

Macrophages
TBEC
| 0.08 | 0.48 | 1.06 | 0.10 | 0.25 | 0.61 | 0.11 | 0.25 | 0.44 |
| --- | --- | --- | --- | --- | --- | --- | --- | --- |
| 0.48 | 0.69 | 1.71 | 1.10 | 0.82 | 1.25 | 0.75 | 0.67 | 0.95 |
| --- | --- | --- | --- | --- | --- | --- | --- | --- |
Subject 1
A20
90kD
A
| 0.48 | 0.91 | 0.66 | 0.51 | 0.54 | 0.96 | 0.51 | 0.97 | 0.94 |
| --- | --- | --- | --- | --- | --- | --- | --- | --- |
| 1.77 | 1.71 | 2.88 | 2.46 | 1.76 | 1.30 | 0.77 | 1.11 | 1.00 |
| --- | --- | --- | --- | --- | --- | --- | --- | --- |
Tollip
28kD
β -actin
42kD
A20
90kD
| 1 | 1.59 | 1.33 | 0.30 | 0.27 | 0.38 | 0.25 | 0.22 | 0.35 |
| --- | --- | --- | --- | --- | --- | --- | --- | --- |
Subject 2
| 0.4 | 0.6 | 0.6 | 0.4 | 0.5 | 0.8 | 0.8 | 0.7 | 0.9 |
| --- | --- | --- | --- | --- | --- | --- | --- | --- |
B
| 2.22 | 3.04 | 2.53 | 1.38 | 1.21 | 0.87 | 0.88 | 0.64 | 0.59 |
| --- | --- | --- | --- | --- | --- | --- | --- | --- |
| 0.45 | 0.76 | 0.77 | 1.25 | 1.95 | 1.98 | 1.67 | 1.88 | 1.67 |
| --- | --- | --- | --- | --- | --- | --- | --- | --- |
Tollip
28kD
β -actin
42kD
Supplementary Figure 2 A, B: Detection of A20, Tollip in human tracheobronchial epithelial cells (TBEC) and alveolar macrophages before and after PAMP stimulation at different time points.

## Slide 3
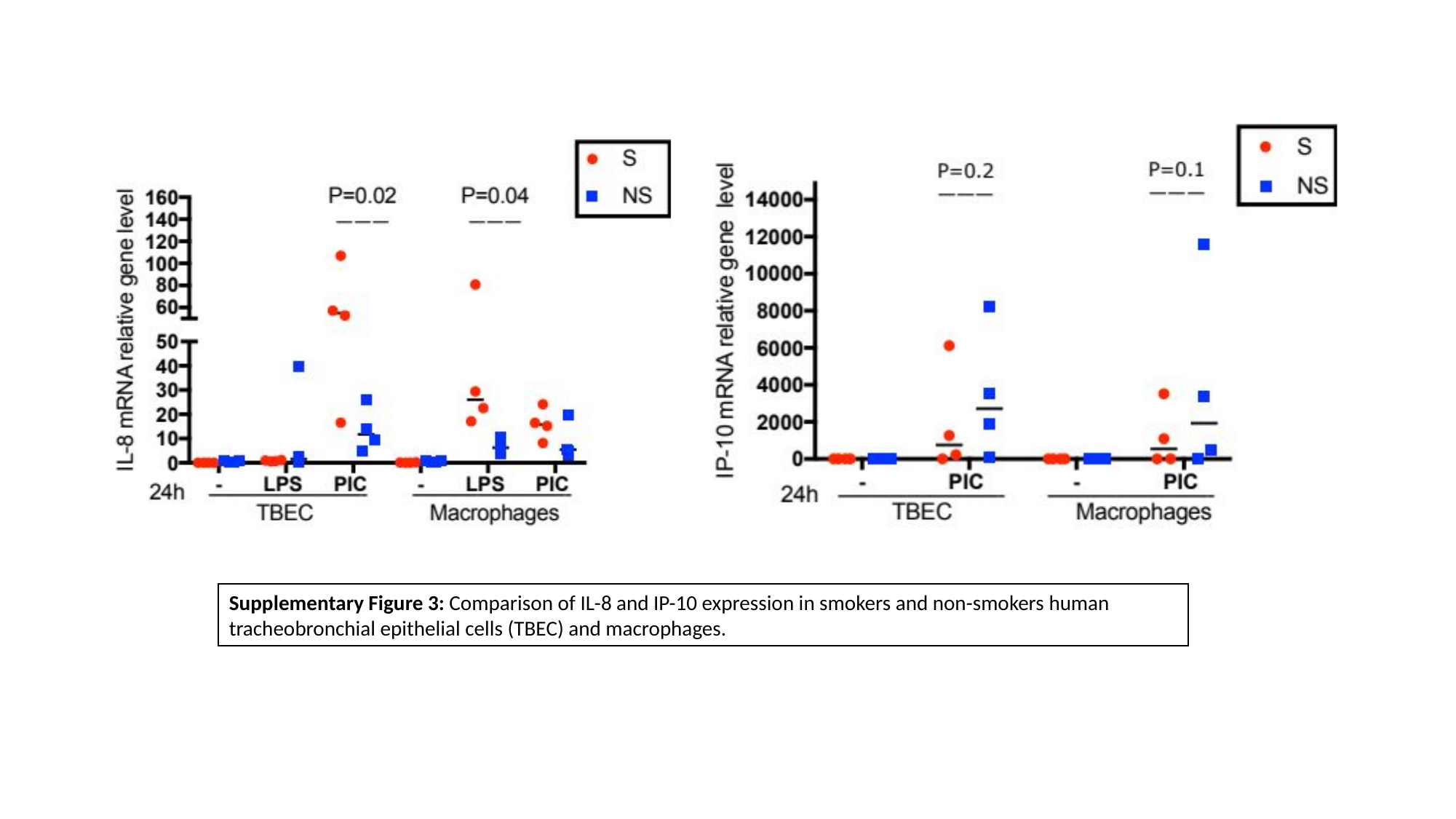

Supplementary Figure 3: Comparison of IL-8 and IP-10 expression in smokers and non-smokers human tracheobronchial epithelial cells (TBEC) and macrophages.

## Slide 4
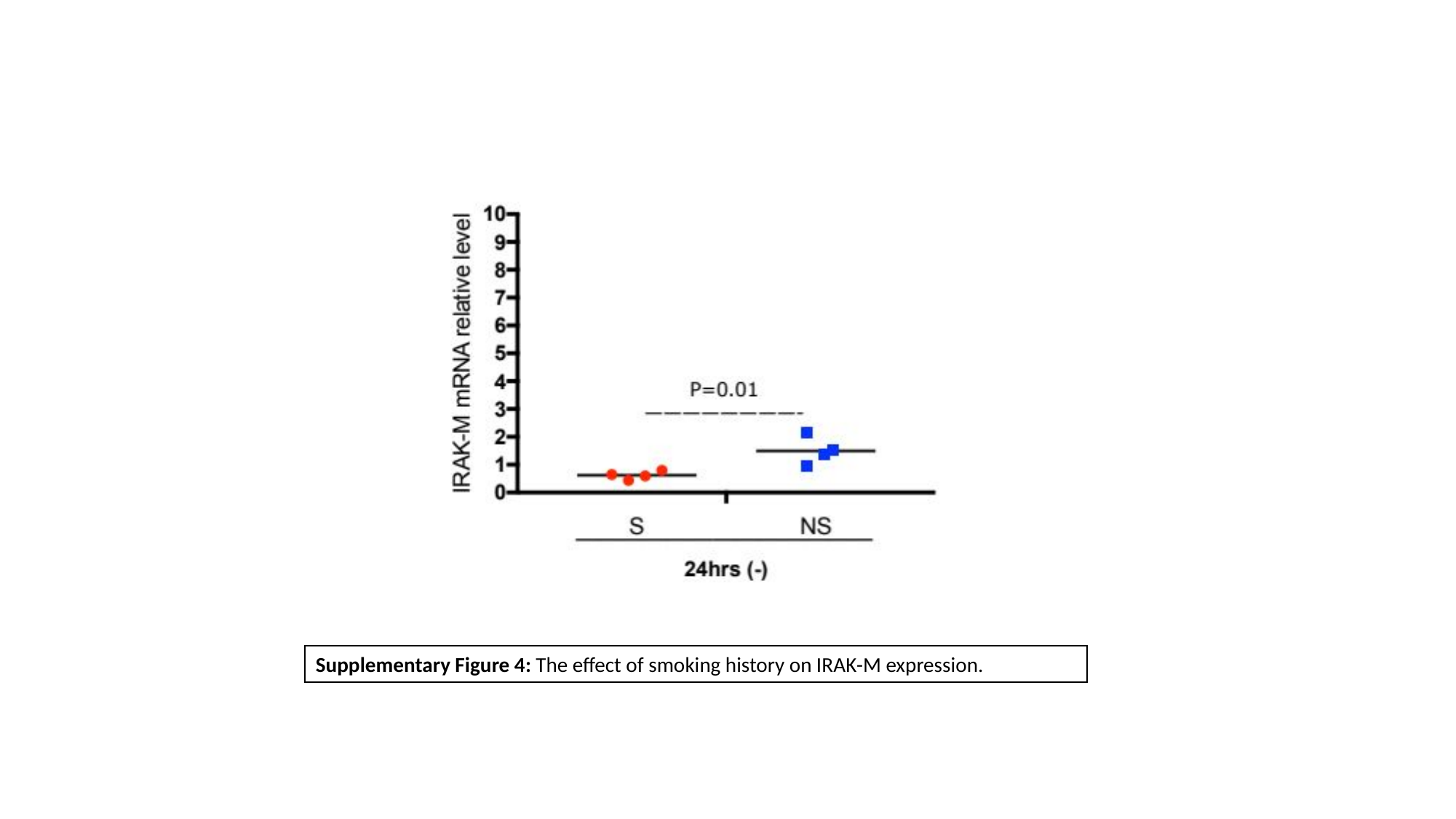

Supplementary Figure 4: The effect of smoking history on IRAK-M expression.

## Slide 5
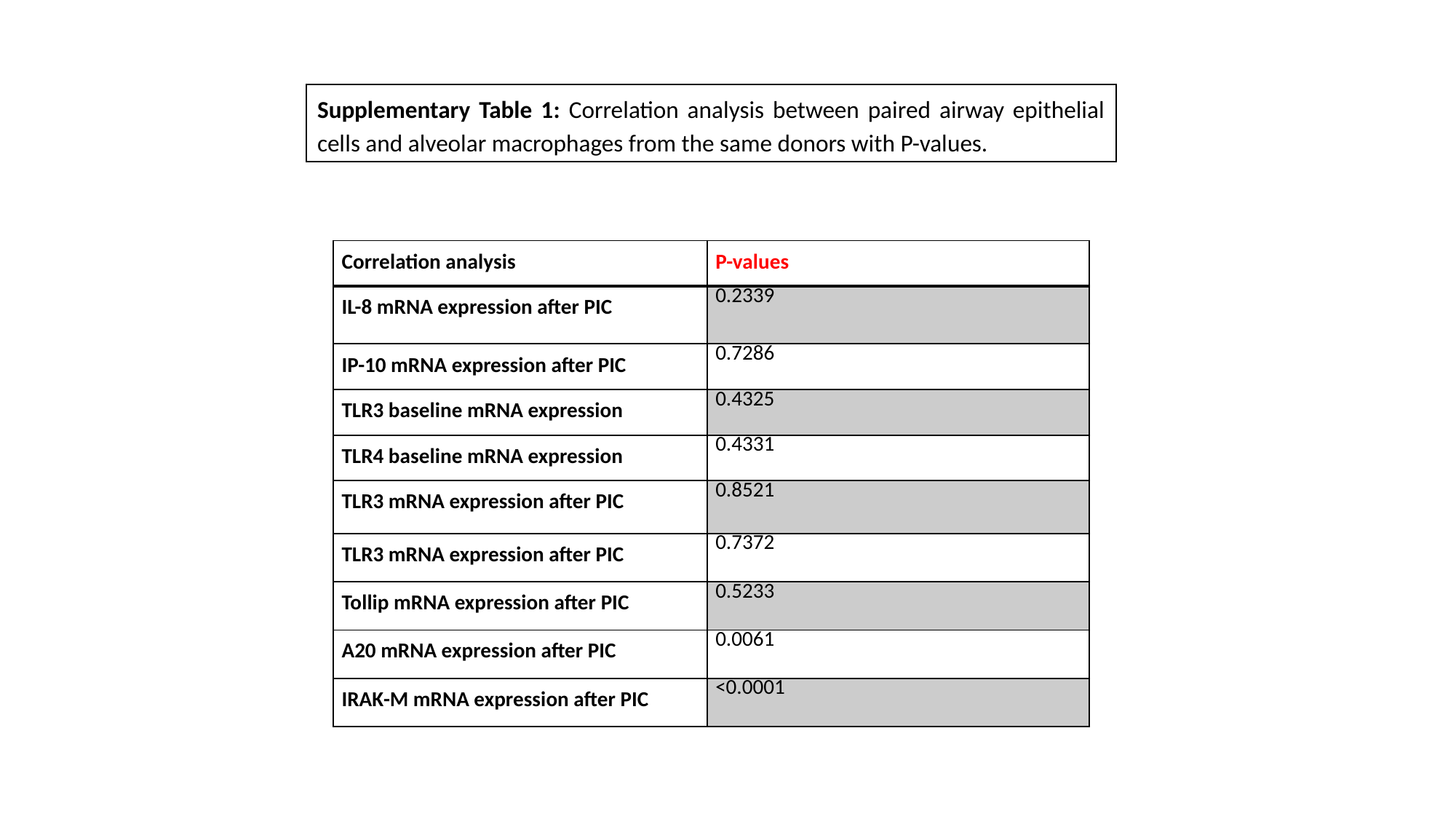

Supplementary Table 1: Correlation analysis between paired airway epithelial cells and alveolar macrophages from the same donors with P-values.
| Correlation analysis | P-values |
| --- | --- |
| IL-8 mRNA expression after PIC | 0.2339 |
| IP-10 mRNA expression after PIC | 0.7286 |
| TLR3 baseline mRNA expression | 0.4325 |
| TLR4 baseline mRNA expression | 0.4331 |
| TLR3 mRNA expression after PIC | 0.8521 |
| TLR3 mRNA expression after PIC | 0.7372 |
| Tollip mRNA expression after PIC | 0.5233 |
| A20 mRNA expression after PIC | 0.0061 |
| IRAK-M mRNA expression after PIC | <0.0001 |
